# Supplementation with the Postbiotic BPL1™-HT (Heat-Inactivated *Bifidobacterium animalis* subsp. Lactis) Attenuates the Cardiovascular Alterations Induced by Angiotensin II Infusion in Mice

**DOI:** 10.3390/antiox14020193

**Published:** 2025-02-08

**Authors:** Mario de la Fuente-Muñoz, Marta Román-Carmena, Sara Amor, Daniel González-Hedström, Verónica Martinez-Rios, Patricia Martorell, Antonio M. Inarejos-García, Reme García Bou, Sonia Guilera-Bermell, Ángel L. García-Villalón, Miriam Granado

**Affiliations:** 1 Departamento de Fisiología, Facultad de Medicina, Universidad Autónoma de Madrid, 28029 Madrid, Spain; mario.delafuente@inv.uam.es (M.d.l.F.-M.); marta.romanc@uam.es (M.R.-C.); sara.amor@uam.es (S.A.); angeluis.villalon@uam.es (Á.L.G.-V.); 2 R&D Department of Functional Extracts, ADM^®^ Valencia, 46740 Carcaixent, Spain; daniel.gonzalezhedstrom@adm.com (D.G.-H.); veronica.martinez@adm.com (V.M.-R.); antonio.inarejos@adm.com (A.M.I.-G.); reme.garcia@adm.com (R.G.B.); sonia.guilera@adm.com (S.G.-B.); 3 Nutrition Archer Daniels Midland (ADM) Health & Wellness, Biopolis S. L. Parc Cientific, Universitat de València, 46980 Paterna, Spain; patricia.martorell@adm.com; 4 CIBER Fisiopatología de la Obesidad y Nutrición, Instituto de Salud Carlos III, 28029 Madrid, Spain

**Keywords:** angiotensin II, hypertension, cardiovascular damage, postbiotic, antioxidant, inflammation

## Abstract

Hypertension is associated with alterations in the composition and diversity of the intestinal microbiota. Indeed, supplementation with probiotics and prebiotics has shown promising results in modulating the gut microbiota and improving cardiovascular health. However, there are no studies regarding the possible beneficial effects of postbiotics on cardiovascular function and particularly on hypertension-induced cardiovascular alterations. Thus, the aim of this study was to analyze the effect of supplementation with the heat-treated *Bifidobacterium animalis* subsp. lactis CECT 8145 strain (BPL1™ HT), a postbiotic developed by the company ADM-Biopolis, on cardiovascular alterations induced by angiotensin II (AngII) infusion in mice. For this purpose, three groups of C57BL/6J male mice were used: (i) mice infused with saline (control); (ii) mice infused with AngII for 4 weeks (AngII); and (iii) mice supplemented with BPL1™ HT in the drinking water (1010 cells/animal/day) for 8 weeks and infused with AngII for the last 4 weeks (AngII + BPL1™ HT). AngII infusion was associated with heart hypertrophy, hypertension, endothelial dysfunction, and overexpression of proinflammatory cytokines in aortic tissue. BPL1™ HT supplementation reduced systolic blood pressure and attenuated AngII-induced endothelial dysfunction in aortic segments. Moreover, mice supplemented with BPL1™ HT showed a decreased gene expression of the proinflammatory cytokine interleukin 6 (*Il-6*) and the prooxidant enzymes NADPH oxidases 1 (*Nox-1*) and 4 (*Nox-4*), as well as an overexpression of AngII receptor 2 (*At2r*) and interleukin 10 (*Il-10*) in arterial tissue. In the heart, BPL1™ HT supplementation increased myocardial contractility and prevented ischemia–reperfusion-induced cardiomyocyte apoptosis. In conclusion, supplementation with the postbiotic BPL1™ HT prevents endothelial dysfunction, lowers blood pressure, and has cardioprotective effects in an experimental model of hypertension induced by AngII infusion in mice.

## 1. Introduction

Cardiovascular diseases are the leading cause of morbidity and mortality worldwide [1], so understanding the underlying mechanisms involved in their development is crucial for improving new therapeutic interventions. A key player in the pathogenesis of cardiovascular disorders is the hormone angiotensin II (AngII), a potent vasoconstrictor peptide produced by the renin–angiotensin system (RAS), which plays a crucial role in the regulation of blood pressure and fluid homeostasis [2]. Moreover, this peptide has been shown to significantly contribute to the development of structural and functional alterations, both at the vascular and at the cardiac level in several cardiovascular diseases, including hypertension [3].

AngII binds to two types of cell membrane receptors: type 1 receptor (AT1R) and type 2 receptor (AT2R), with most of the biological actions like vasoconstriction, cell growth, apoptosis, inflammation and oxidative stress induction being mediated by its binding to the AT1R [4,5]. These effects are associated with the development of tissue damage, ultimately leading to several cardiovascular alterations such as hypertension, arrhythmias, cardiac hypertrophy, fibrosis, or heart failure [6].

In the blood vessels, AngII induces vascular damage through a complex and multifaceted process that involves the activation of various signaling pathways and the interplay of numerous factors, with inflammation and oxidative stress emerging as central mechanisms [3,7]. One of the hallmarks of AngII-induced vascular damage is its ability to promote the migration and recruitment of leukocytes into the arterial subendothelial space. This process is mediated through a sequential and coordinated cascade of leukocyte–endothelial cell adhesive interactions, involving an array of cell adhesion molecules and the generation and release of chemoattractants, such as the monocyte chemoattractant protein-1 (MCP-1), that activate and guide leukocytes to the sites of emigration [3]. This inflammatory response is closely linked to the impairment of endothelial function, leading to increased permeability, the increased production of proinflammatory cytokines and growth factors [8], and altered vascular tone due to the decreased bioavailability of nitric oxide [9].

In the heart, chronic exposure to AngII can lead to sustained increases in afterload, placing undue stress on the myocardium [10]. This sustained pressure overload triggers maladaptive remodeling processes, including cardiomyocyte hypertrophy, interstitial fibrosis, and impaired diastolic function [6]. The hypertrophy of cardiomyocytes, accompanied by an increase in the deposition of the collagen-rich extracellular matrix, results in a stiffened and less compliant myocardium, which compromises its ability to fill and relax properly during diastole [11]. Additionally, AngII can directly induce cardiomyocyte apoptosis and necrosis, further exacerbating the structural and functional impairments within the heart [3]. The loss of viable cardiomyocytes and the disruption of the myocardial architecture induced by chronic AngII exposure ultimately lead to a decline in cardiac contractility and overall cardiac performance [12].

In addition to the deleterious direct effects of AngII on the cardiovascular system, AngII can also induce structural and functional alterations on the heart and blood vessels through the activation of the Nox1 and Nox2 NADPH oxidase subunits, which derives in an increased production of reactive oxygen species, such as superoxide anion [13]. Indeed, besides the conventional pharmacological treatments, antioxidants have emerged as potential candidates for the treatment and/or prevention of the cardiovascular diseases that are associated with increased AngII levels, particularly hypertension [14]. In this regard, several antioxidants such as Vitamins C and E [15] or n-acetylcysteine [16,17] have been demonstrated to be effective in reducing blood pressure in experimental animals. Moreover, certain antioxidants such as coenzyme Q10 show antihypertensive effects not only in rats [18] but also in humans [19].

Other compounds that have recently been proposed for the management of cardiovascular diseases are probiotics [20]. Probiotics have been proven to be effective in several cardiovascular disorders such as chronic heart failure, atherosclerosis, or hypertension [21] due to their antioxidant, anti-inflammatory, and hypocholesterolemic effects and thanks to their ability to regulate gut microbiota function and metabolism [20,22]. Probiotics are defined as live microorganisms that, when administered in adequate amounts, confer several health benefits to the host. They include bacteria from different genera such as Bifidobacterium, Lactobacillus, Enterococcus, Lactococcus, Pediococcus, Leuconostoc, Propionibacterium, Bacillus, Escherichia coli, or Streptococcus and yeasts such as Saccharomyces [23]. Among probiotics, Bifidobacterium spp. strains are reported to be effective in reducing some of the classical risk factors for cardiovascular diseases (e.g., obesity, hyperlipidemia, diabetes) by reducing oxidative stress and by improving immunomodulation and glucose and lipidic metabolism [24]. However, one of the main drawbacks for the use of probiotics in therapeutics is their possible side effects, especially in patients with underlying medical conditions [20,25]. Moreover, another limitation is that they need to meet several requirements to be used for human health, such as remaining viable after the action of gastric juice and bile salts, promoting the growth of beneficial microorganisms and avoiding the growth of pathogenic bacteria, and exerting adhesive properties to the intestine surface after passing through the chemical barrier. A promising alternative to probiotics could be the use of postbiotics, which, in 2021, were defined by the International Scientific Association for Probiotics and Prebiotics (ISAPP) as “a preparation of inanimate microorganisms and/or their components that confers a health benefit on the host” [26]. This definition of postbiotic implies the presence of inanimate intact cells of a well-defined microorganism or combination of microorganisms or, at least, some of their structural components such as cell walls, microbial fragments, etc. The interest in postbiotics relies in their safety and stability because, unlike probiotics which require specific storage conditions to avoid their inactivation, postbiotics are more resistant and can be more easily incorporated into various products, including pharmaceuticals, functional foods, and dietary supplements. Furthermore, as they are non-viable cells, there is no risk of adverse effects associated with live microorganisms. However, knowledge regarding the role of postbiotics in cardiovascular health is currently limited. Indeed, there is only one published clinical study on the subject reporting the antihypertensive effects of supplementation with heat-treated Lactobacillus reuteri ADR-3 in patients with type 2 diabetes [27].

In this study, we aimed to study the possible beneficial cardiovascular effects of supplementation with the postbiotic *Bifidobacterium animalis* subsp. lactis CECT 8145 inactivated by heat (BPL1™ HT). Previous studies have shown that *Bifidobacterium animalis* subsp. lactis CECT 8145 (BPL1™) and its heat-treated version are able to reduce fat content, modulate lipid metabolism, and protect against oxidative stress in a Caenorhabditis elegans model [28]. Additionally, the beneficial effects of BPL1™ and BPL1™ HT have been proven not only in murine studies with Zücker rats [29] or Wistar rats fed with a cafeteria diet [30], but also in clinical studies with human obese volunteers [31]. Moreover, the consumption of BPL1™ HT-enriched seafood sticks has been reported to significantly reduce systolic blood pressure in women with abdominal obesity after 21 weeks of supplementation [32]. Nevertheless, the mechanisms involved in such antihypertensive effects are unknown. Thus, the aim of this study was to analyze whether supplementation with the postbiotic BPL1 HT™ also exerts beneficial cardiovascular effects in an experimental model of hypertension induced by AngII infusion in mice and, if that is the case, to elucidate the possible mechanisms involved in such cardioprotective effects.

## 2. Materials and Methods

### 2.1. Animals

Twenty-four 16-week-old C57BL/6J male mice were housed three per cage and maintained in climate-controlled quarters with a 12 h light cycle under controlled temperature conditions (22–24 °C) and humidity (50–60%). Experiments were performed with the approval of the Ethical Committee of the Community of Madrid (Madrid, Spain) (PROEX 282.2-22).

Mice were fed ad libitum for 8 weeks with chow. Heat-treated strain BPL1™ HT was provided by the company ADM/Biopolis and administered in the drinking water (10^10^ cells/animal/day). After four weeks of treatment, Alzet osmotic minipumps (Alza Corp., Cupertino, CA, USA; 2004 model) were implanted subcutaneously under isoflurane anesthesia (2%) to infuse either AngII (1.44 mg/Kg/day) or saline for 28 days (infusion rate = 0.25 µL/h). Afterwards, the animals were divided into three experimental groups: (i) mice infused with saline (control; *n* = 7); (ii) mice infused with AngII (AngII; *n* = 8); and (iii) mice infused with AngII and supplemented with BPL1™ HT (AngII + HTBPL1; *n* = 8).

Body weight and food intake were monitored once a week over the treatment period. After 8 weeks of treatment, all animals were sacrificed by decapitation after an injection of sodium pentobarbital (100 mg/kg). After sacrifice, the heart and the aorta were dissected for the functional and molecular analysis. The trunk blood was collected in tubes containing EDTA (1.5 mg/mL) and centrifuged at 3000 rpm for 20 min to obtain the plasma.

### 2.2. Measurement of Systolic Blood Pressure in Conscious Mice by Plethysmography

Systolic blood pressure was measured twice every week by transmission photoplethysmography using a Blood Pressure Analysis System (BP-2000; Visitech Systems, Apex, NC, USA). For this purpose, all mice were accustomed to the device at least three days before the measurements. Briefly, an occlusion cuff was placed at the base of the tail of the mice in a prewarmed plate at 37 °C. Every session consisted of 30 inflation–deflation cycles.

### 2.3. Plasma Measurements

Plasma concentrations of Ang 1–7 were measured by an ELISA kit (ABclonal, Woburn, MA, USA) following the manufacturer’s instructions. Sensitivity and intra-assay variations were 2.2 pg/mL and 6.4%, respectively.

Malondialdehyde (MDA) was quantified by HPLC according to Más-Bargues et al. [33]. For this purpose, an HPLC equipment Shimadzu Nexera XR HPLC 70 MPa coupled to a photodiode array detector (SPD-M40, Izasa Scientific, Alcobendas, Spain) was used. An octadecyl silane column Zorbax Eclipse Plus C18 (250 mm, 4.6 mm, 5 µL) and a precolumn (Agilent Technologies, Las Rozas, Madrid, Spain) were used. The quantification of MDA was performed according to the protocol described by Wong et al. [34] at a wavelength of 532 nm.

### 2.4. Experiments of Vascular Reactivity

After aorta dissection, 2 mm segments were mounted in a 4 mL organ bath for the recording of the isometric tension using a PowerLab data acquisition system (ADInstruments, Colorado Springs, CO, USA). An optimal passive tension of 1 g was applied and, after 60–90 min equilibration, potassium chloride (KCl 100 mM, Merck Millipore, Burlington, MA, USA) was added to the organ bath. Segments unable to contract at least 0.3 g in response to KCl were discarded.

Vasodilation studies were performed in thoracic aorta segments precontracted with the thromboxane analog U46619 10^−7.5^ M (Sigma-Aldrich, St. Louis, MO, USA), in which dose–response curves to sodium nitroprusside (NTP; 10^−9^–10^−5^ M) and acetylcholine (Ach; 10^−9^–10^−4^ M) were performed. Some aorta segments were pre-incubated with the antioxidant apocynin (10^−6^ M) for 30 min before the dose–response curve in response to Ach. The % of relaxation in response to each Ach concentration was calculated considering the maximum relaxation (Emax) in response to NTP (10^−5^ M) as 100%.

### 2.5. Experiments of Heart Perfusion

Immediately after sacrifice, the heart was removed, cannulated through the aorta, and mounted in a heart perfusion system (Langendorff) to be retrogradely perfused with an oxygenated Krebs–Henseleit solution (115 mM NaCl, 4.6 mM KCl, 1.2 mM KH_2_PO_4_, 1.2 mM MgSO_4_, 2.5 mM CaCl_2_, 25 mM NaHCO_3_ and 11 mM glucose) with the help of a peristaltic pump (Gilson, Middleton, WI, USA). The pH (7.4) and the flow rate were maintained at a constant value (12–17 mL)/min by providing a basal perfusion pressure of around 70 mmHg. The changes in the coronary perfusion pressure were measured through a lateral pressure transducer connected to the perfusion cannula (Statham Instruments, Los Angeles, CA, USA). To measure the intraventricular pressure, a latex balloon inserted into the left ventricle was connected to another pressure transducer. As an index of heart contractility, the intraventricular pressure was used to calculate the heart rate and the first derivative of the left intraventricular pressure vs. time (dP/dt). After 30 min of heart perfusion with a constant flow, global ischemia was induced by stopping the perfusion pump for 30 min. Then, the hearts were re-perfused for 45 min. Data of cardiac function were recorded using the PowerLab/8e data acquisition system (ADInstruments, Colorado Springs, CO, USA). After ischemia–reperfusion (IR), all hearts were collected and stored at −80 °C for further analysis.

### 2.6. Quantitative qRT-PCR

Total RNA was extracted from aortic and myocardial tissue using the Tri-Reagent protocol [35]. Afterwards, 1 μg of total RNA was reverse-transcribed into cDNA using a high-capacity cDNA RT kit (Applied Biosystems, Foster City, CA, USA). The gene expression of interleukin-6 (Il-6) (Mm00446190_m1), interleukin-1 beta (Il-1β) (Mm00434228_m1), interleukin-10 (Il-10) (Mm01288386_m1), tumor necrosis factor-alpha (Tnf-α) (Mm00443258_m1), monocyte chemoattractant protein (Mcp-1) (Mm00441242_m1), glutathione reductase (Gsr) (Mm00439154_m1), NADPH oxidase-4 (Nox-4) (Mm00479246_m1), NADPH oxidase-1 (Nox-1) (Mm00549170_m1), superoxide dismutase 1 (Sod-1) (Mm01344233_g1), Glutathione Peroxidase 3 (Gpx3) (Mm00492427_m1), angiotensin receptor type-1 (At1r) (Mm00616371_m1), and angiotensin receptor type-2 (At2r) (Mm01341373_m1) was assessed by quantitative real-time PCR (qPCR), which was performed using assay on-demand kits (Applied Biosystems, Foster City, CA, USA). For that purpose, the DNA was amplified using TaqMan Universal PCR Master Mix (Applied Biosystems, Foster City, CA, USA) in Step One System equipment (Applied Biosystems, Foster City, CA, USA). The Ct values were normalized to the Ct values of the housekeeping gene Hypoxanthine Phosphoribosyltransferase 1 (Hprt1) (Mm03024075_m1). Relative expression was determined by the 2^−ΔΔCT^ method as previously described [36]. Data were expressed considering the expression of the control group as 100%.

### 2.7. Protein Quantification by Western Blot

Cardiac tissue (100 mg) was homogenized in RIPA buffer (500 μL) and centrifugated for 20 min at 14,000 rpm (4 °C). The total protein content was measured in the supernatant by the Bradford assay (Sigma-Aldrich, St. Louis, MO, USA). Total proteins were loaded in 10% acrylamide SDS gels (20 μg/well) and separated by electrophoresis. Subsequently, proteins were transferred to polyvinylidene difluoride (PVDF) membranes (Bio-Rad, Hércules, CA, USA). Ponceau red dyeing (Sigma-Aldrich, St. Louis, MO, USA) was used to assess the transfer efficiency. After blocking the membranes with tris-buffered saline (TBS) containing 5% (*w*/*v*) non-fat dried milk, they were incubated with the primary antibody for Caspase 8 (1:2000; #PA5-20118; Thermo Scientific, Waltham, MA, USA). Then, membranes were washed four times with TBS-T (10 min/washing) and incubated with the secondary antibody conjugated with peroxidase (1:2000; Pierce, Rockford, IL, USA). Peroxidase activity was detected by the BioRad Molecular Imager ChemiDoc XRS System (Hercules, CA, USA) and quantified by densitometry using the software Image J (vs 1.54f). The average protein expression levels for each experimental group was calculated as a percentage of the average expression of the control group.

### 2.8. Histological Analysis

Aorta and heart tissues were fixed (4% paraformaldehyde) and dehydrated. Afterwards, they were embedded in paraffin and sectioned in 5 μm slices. Arterial sections were stained with hematoxylin/eosin (H/E) to assess the thickness of the tunica media. The arterial production of superoxide anions was assessed by the dihydroethidium (DHE, Invitrogen Life Technologies, Carlsbad, CA, USA) staining. For that purpose, DHE was dissolved in DMSO (2.5 mM), added to each slice and incubated for 30 min at 37 °C in a dark and humidified chamber. Then, the slices were mounted with Vectashield (Newark, CA, USA). Finally, a confocal microscope (Leica TCS SP2 equipped with a krypton/argon laser, ×40 objective; Leica Microsystems, Lane Cove West, Australia) was used to obtain the images with a fluorescence 563 nm long-pass filter. The mean fluorescence density was analyzed for each section; 3–5 slices per animal from each experimental group were analyzed. Data were expressed as a percentage of the average fluorescent signal in sections from control mice.

Apoptosis of cardiomyocytes after IR was assessed by TUNEL staining according to the manufacturer’s protocol (Click-iTTM Plus TUNEL Assay; Invitrogen, Carlsbad, CA, USA) in a confocal microscope (Leica TCS SP5 equipped with 40× oil objective; Leica Microsystems S.L.U.) with a 519 nm long-pass filter. 3–4 images of different regions were sampled per animal. Quantitative analysis was performed with the ImageJ software. Results were expressed as the percentage of cells with fragmented DNA vs. the total number of cells. Data from the different experimental groups was expressed as a percentage of the signal obtained in arteries from control mice.

### 2.9. Statistical Analysis

Statistical analysis was performed by one-way ANOVA followed by Bonferroni’s post hoc test using the GraphPad Prism V.70.4 statistical software (GraphPad Software, Inc., San Diego, CA, USA). Data are expressed as mean values ± standard error of the mean (SEM). *p* < 0.05 was considered statistically significant.

## 3. Results

### 3.1. Systolic Blood Pressure

**Figure 1:**
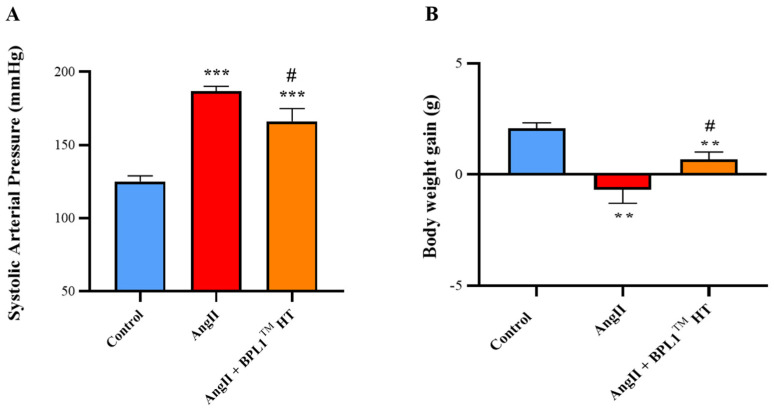
Systolic blood pressure (**A**) and body weight gain (**B**) in mice infused with saline (control), mice infused with AngII (AngII), and mice infused with AngII and supplemented with HTBPL-1 (AngII + HTBPL1). Values are represented as mean value ± SEM; *n* = 7–8 mice/group. ** *p* < 0.01 vs. control; *** *p* < 0.001 vs. control; # *p* < 0.001 vs. AngII.

As shown in Figure 1A, infusion of AngII for four weeks induced a significant increase in systolic blood pressure and this increase was significantly reduced by BPL1™ HT treatment.

### 3.2. Body Weight

There were no differences in body weight gain between the animals from the different experimental groups during the first four weeks of treatment. However, after the implantation of the osmotic minipumps (week four), AngII infusion was associated with a significant decrease in body weight gain that was prevented by BPL1™ HT supplementation (Figure 1B). These effects were not associated with changes in solid or liquid daily intake.

### 3.3. Organ Weights

As shown in Table 1, neither AngII infusion nor BPL1™ HT supplementation induced significant changes in the weights of spleen, liver, brain, skeletal muscles (soleus and gastrocnemius) and adipose tissue depots (lumbar, epididymal, retroperitoneal, and interscapular). However, mice infused with AngII showed a significant increase in the weight of the heart and in the weight of the adrenal glands that was attenuated, in the case of the adrenal glands, by BPL1™ HT supplementation. Moreover, treatment with BPL1™ HT significantly reduced the weight of the kidneys in mice infused with AngII.

### 3.4. Vascular Reactivity

**Figure 2:**
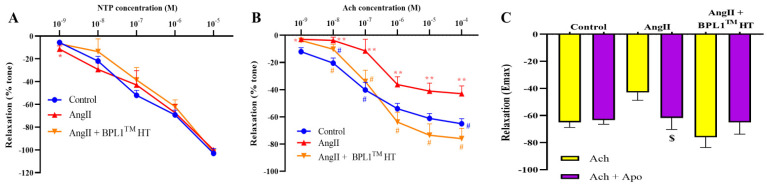
Relaxation of thoracic aortic segments to sodium nitroprusside (NTP) (10^−9^–10^−5^ M) (**A**) and to acetylcholine (ACh) (10^−9^–10^−4^ M) (**B**); Emax relaxation to acetylcholine in the presence/absence of apocynin (ACh/ACh + Apo) (10^−6^ M) (**C**) of mice infused with saline (control), mice infused with AngII (AngII), and mice infused with AngII and supplemented with BPL1™ HT (AngII + BPL1™ HT). One aortic ring from each mouse was used for each condition (*n* = 7–8 samples/group). Values are represented as mean value ± SEM;. * *p* < 0.05 vs. control; ** *p* < 0.01 vs. control; # *p* < 0.05 vs. AngII; $ *p* < 0.05 vs. Ach of its experimental group.

The endothelium-independent relaxation of aortic rings in response to sodium nitroprusside (NTP) was not modified by AngII infusion or by BPL1™ HT supplementation (Figure 2A). However, the endothelium-dependent relaxation in response to acetylcholine (Ach) was significantly reduced in the aorta segments from mice infused with AngII compared to aorta segments from control mice, with this reduction being totally prevented by treatment with BPL1™ HT (Figure 2B). To analyze the possible mechanism involved in AngII-induced endothelial dysfunction, some aorta segments were preincubated with the antioxidant apocynin (10^−6^ M) before performing the dose–response curve to Ach. Pre-incubation with apocynin did not modify the vasodilator response to Ach in aorta segments from control mice nor in aorta segments from mice infused with AngII and supplemented with BPL1™ HT (Figure 2C). However, aorta segments from untreated mice infused with AngII showed a significant improvement in Ach-induced relaxation in the presence of the antioxidant.

### 3.5. Thickness of Arterial Wall and Gene Expression of Inflammatory Markers in Arterial Tissue

**Figure 3:**
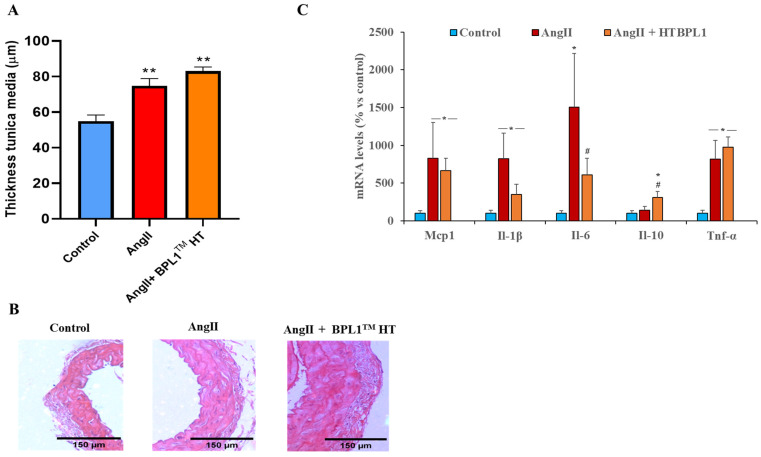
Thickness of the tunica media (**A**) and representative images of aorta sections stained with hematoxylin–eosin (**B**). The scale bar is equivalent to 150 μm. Gene expression of monocyte chemotactic protein-1 (Mcp-1), interleukin-1β (Il-1β), interleukin-6 (Il-6), interleukin-10 (Il-10), and tumor necrosis factor α (Tnf-α) (**C**) in mice aorta infused with saline (control), infused with AngII (AngII), and infused with AngII and supplemented with BPL1™ HT (AngII + BPL1™ HT). Data are represented as mean value ± SEM; *n* = 7–8 mice/group. For the assessment of tunica media thickness, four sections of four different mice from each experimental group were used. * *p* < 0.05 vs. control; ** *p* < 0.05 vs. control; # *p* < 0.05 vs. AngII.

AngII infusion for four weeks induced an increase in the thickness of the aorta’s tunica media that was not prevented by BPL1™ HT treatment (Figure 3A,B). In addition, as shown in Figure 3C, mice infused with AngII showed an upregulation in the gene expression of the proinflammatory cytokines Il-1β, Il-6, and Tnf-α and the chemokine Mcp-1 in arterial tissue. These changes were not prevented by BPL1™ HT treatment, except for the mRNA levels of Il-6, which were significantly reduced compared to hypertensive non-treated animals. Finally, the arterial gene expression of the anti-inflammatory cytokine Il-10 was unchanged between the controls and untreated AngII-infused mice, but it was overexpressed in mice infused with AngII and supplemented with BPL1™ HT.

### 3.6. Superoxide Anion and Oxidative Stress Markers in Arterial Tissue

**Figure 4:**
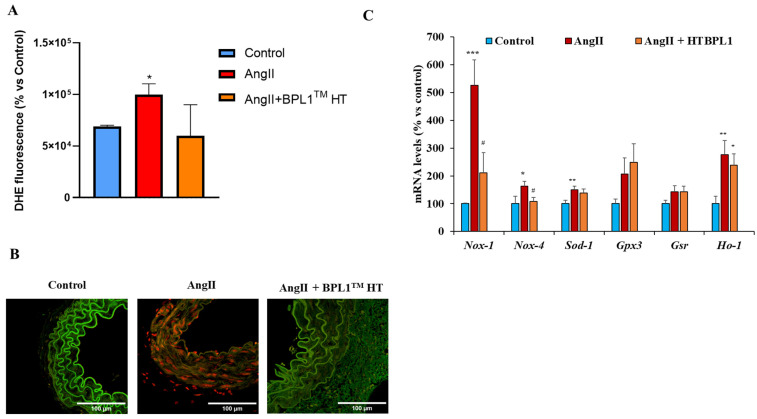
Quantification of reactive oxygen species (**A**) and representative images of aorta sections stained with dihydroethidium (**B**). The scale bar is equivalent to 100 μm. Gene expression of NADPH oxidase 1 (Nox-1), NADPH oxidase 4 (Nox-4), super oxide dismutase 1 (Sod-1), glutathione peroxidase 3 (Gpx-3), glutathione reductase (Gsr), and hemoxigenase 1 (Ho-1) (**C**) in aorta of mice infused with saline (control), mice infused with AngII (AngII), and mice infused with AngII and supplemented with BPL1™ HT (AngII + BPL1™ HT). Values are represented as the mean ± S.E.M (*n* = 7–8 mice/experimental group). For the DHE staining, four sections of four different mice from each experimental group were used. * *p* < 0.05 vs. control; ** *p* < 0.01 vs. control; *** *p* < 0.001 vs. control; # *p* < 0.05 vs. AngII.

Figure 4A and B show the content of superoxide anions in arterial tissue from mice from the different experimental groups. Results indicate that BPL1™ HT supplementation prevented the significant increase in superoxide anion production in arterial tissue produced after AngII infusion. Moreover, BPL1™ HT reversed the upregulation of the pro-oxidant enzymes Nox-1 and Nox-4 mRNA in the arterial tissue of AngII-infused mice (Figure 4C). However, supplementation with BPL1™ HT did not modify the mRNA levels of other antioxidant enzymes such as Ho-1, Gpx3, Gsr, and Sod1.

### 3.7. Cardiac Function and Cardiomyocyte Apoptosis After Ischemia–Reperfusion

**Figure 5:**
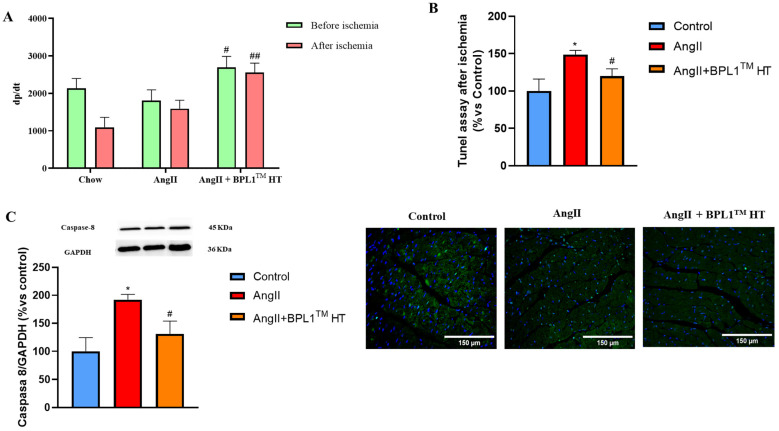
Heart contractility measured before and after ischemia–reperfusion (**A**), quantification of cardiomyocyte apoptosis in ischemic hearts analyzed by TUNEL assay, and representative images of heart sections stained with TUNEL staining; (**B**) protein content of Caspase 8 (**C**) in ischemic hearts of mice infused with saline (control), mice infused with AngII (AngII), and mice infused with AngII and supplemented with BPL1™ HT (AngII + BPL1™ HT). Values are represented as the mean ± S.E.M (*n* = 7–8 mice/group). The scale bar is equivalent to 100 μm. For the analysis of Caspase 8 protein content, 3–4 samples per experimental group were used. * *p* < 0.05 vs. control; # *p* < 0.05 vs. AngII; ## *p* < 0.01 vs. AngII.

Supplementation with BPL1™ HT increased heart contractility (dP/dt) before and after subjecting the hearts to 30 min of ischemia and 15 min of reperfusion (IR) (Figure 5A). Moreover, supplementation with BPL1™ HT attenuated the AngII-induced increase in cardiac apoptosis measured by TUNEL (Figure 5B) and the AngII-induced overexpression of the proapoptotic protein caspase-8 in myocardial tissue (Figure 5C).

### 3.8. Gene Expression of Inflammatory and Oxidative Stress Markers in Myocardial Tissue

**Figure 6:**
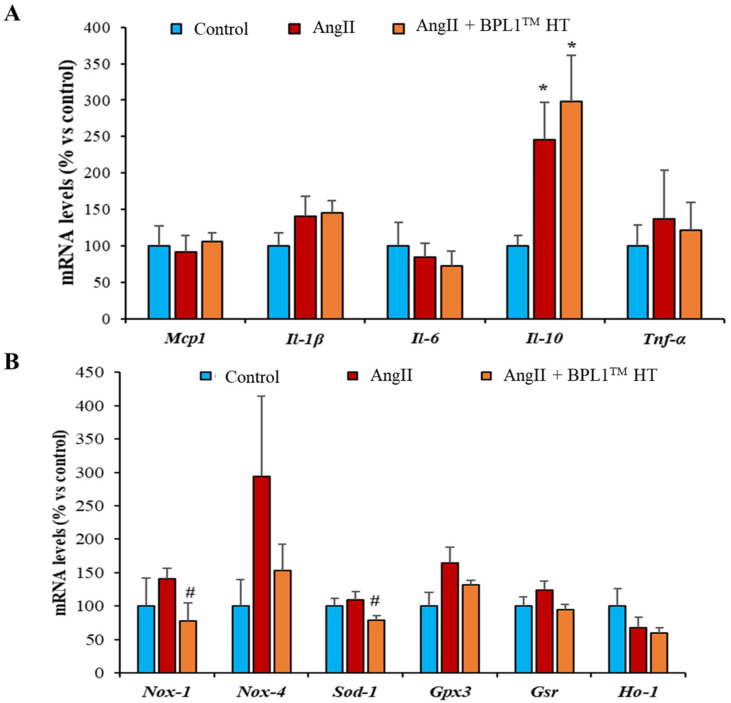
Gene expression of monocyte chemotactic protein-1 (Mcp-1), interleukin-1β (Il-1β), interleukin-6 (Il-6), interleukin-10 (Il-10), and tumor necrosis factor α (Tnf-α) (**A**); gene expression of NADPH oxidase 1 (Nox-1), NADPH oxidase 4 (Nox-4), super oxide dismutase 1 (Sod-1), glutathione peroxidase 3 (Gpx-3), glutathione reductase (Gsr), and hemoxigenase 1 (Ho-1) (**B**) in the heart of mice infused with saline (control), mice infused with AngII (AngII), and mice infused with AngII and supplemented with BPL1™ HT (AngII + BPL1™ HT). The values are represented as the mean ± S.E.M (*n* = 7–8 samples/experimental group by duplicate) and expressed as a percentage vs. control; * *p* < 0.05 vs. control; # *p* < 0.05 vs. AngII.

No significant changes were found in the gene expression of any of the inflammatory markers analyzed except for a significant overexpression of II-10 in the cardiac tissue of AngII-infused animals regardless of the supplementation with BPL1™ HT (Figure 6A). In terms of oxidative stress markers, a significant decrease in the mRNA levels of the enzymes Nox-1 and Sod-1 was observed in the arterial tissue of hypertensive mice supplemented with BPL1™ HT compared to non-supplemented animals infused with AngII (Figure 6B).

### 3.9. Plasma Levels of Ang 1–7 and Gene Expression of AT1R and AT2R in Arterial and Cardiac Tissues

**Figure 7:**
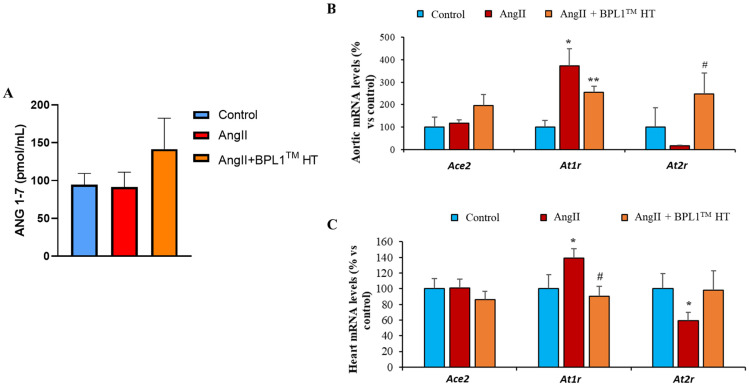
Plasma levels of angiotensin-(1–7) (**A**) and gene expression of angiotensin converting enzyme 2 (ACE2), angiotensin II receptor type 1 (At1r), and angiotensin II receptor type 2 (At2r) in the aorta (**B**) and in the heart (**C**) of mice infused with saline (control), mice infused with AngII (AngII), and mice infused with AngII and supplemented with BPL1™ HT (AngII + BPL1™ HT). The values are represented as the mean ± S.E.M (*n* = 7–8 samples/experimental group) and expressed as a percentage vs. control; * *p* < 0.05 vs. control; ** *p* < 0.01 vs. control; # *p* < 0.05 vs. AngII.

As shown in Figure 7A, the circulating levels of Ang 1–7 were unchanged among experimental groups. Likewise, there were no differences between groups in the mRNA levels of Ace2 in myocardial tissue (Figure 7C). However, AngII infusion induced an overexpression of At1r both in the aorta and in the heart that was prevented after supplementation with BPL1™ HT (Figure 7B,C). Moreover, treatment with the postbiotic BPL1™ HT prevented the AngII-induced downregulation of At2r mRNA levels in arterial tissue (Figure 7B).

### 3.10. Plasma Levels of Malondialdehyde (MDA)

**Figure 8:**
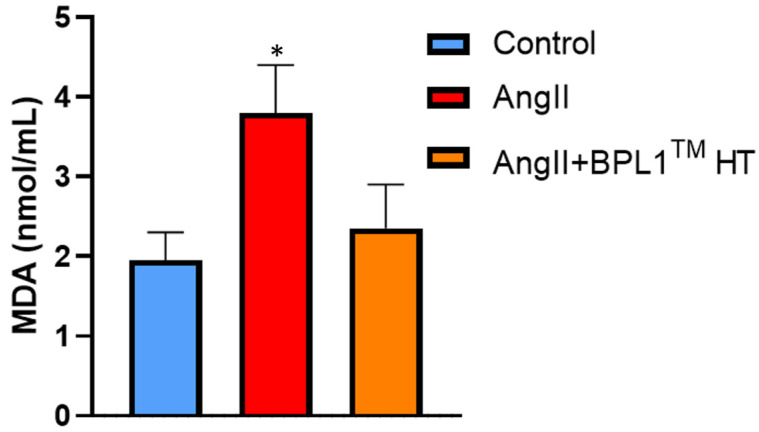
Malondialdehyde (MDA) plasma levels measured by high-performance liquid chromatography (HPLC) of mice infused with saline (control), mice infused with AngII (AngII), and mice infused with AngII and supplemented with BPL1™ HT (AngII + BPL1™ HT). The values are represented as the mean ± S.E.M (*n* = 7–8 samples/experimental group). * *p* < 0.05 vs. control.

Figure 8 shows the circulating levels of MDA in the plasma of control mice, untreated AngII-infused mice, and AngII-infused mice supplemented with BPL1™ HT.

The plasma concentrations of MDA were significantly increased in untreated hypertensive mice. Interestingly, there were no differences between the circulating levels of MDA between control mice and hypertensive mice treated with BPL1™ HT.

## 4. Discussion

The results of this study indicate, for the first time, that supplementation with the heat-treated strain *Bifidobacterium animalis* subsp. lactis BPL1™-HT (CECT 8145) h) attenuates AngII-induced cardiovascular damage.

One of the most relevant findings is that mice infused with AngII for four weeks and supplemented with the postbiotic BPL1™ HT showed a significant decrease in systolic blood pressure (SBP), pointing to an antihypertensive effect of the postbiotic. As previously described [37], the increased SPB in AngII-infused animals resulted in a significant decrease in body weight gain, with this effect being attenuated by BPL1™ HT supplementation. Since body weight loss in rodents is associated with stress and alterations in animal well-being [38], the greater body weight gain in hypertensive animals supplemented with the postbiotic clearly indicates an improvement in their metabolic state and in their general health status. Our results agree with those described by Companys et al., who reported a significant reduction in SBP in women with abdominal obesity after supplementation with BPL1™ HT-enriched seafood for twelve weeks [32]. Likewise, Hsieh et al. reported the antihypertensive effects of another postbiotic (heat-treated L.reuteri ADR-3) in patients with type 2 diabetes [27]. The positive effect of BPL1™ HT reducing SBP in mice infused with AngII seem to be mediated, at least in part, through their antioxidant effects, which are evidenced by the downregulation of the mRNA levels of the prooxidant enzymes Nox-1 and Nox-4 and by the significant reduction in superoxide anion production in arterial tissue. Furthermore, untreated AngII-infused mice showed a significant increase in the circulating levels of MDA, while the plasma levels of MDA in AngII-infused mice supplemented with BPL1™ HT were not different from those of control animals. In agreement with these results, it is reported that the health benefits exerted by other postbiotics are also associated with decreased plasma concentrations of MDA [39].

In addition, treatment with BPL1™ HT given to AngII-infused mice significantly reduced the mRNA levels of Il-6 and upregulated the gene expression of Il-10 in arterial tissues, which also demonstrates its anti-inflammatory activity at the vascular level. These results agree with those reported in a very recent study in which the beneficial effects of a postbiotic based on a mixture of inanimate bacteria (L. plantarum, L. reuteri, L. casei, L. rham-nosus, L. mucosae, L. fermentum, L. delbrueckii, and L. brevis, along with B. bifidum, B. longum, and B. infantis) isolated from the breast milk on dextran sulfate sodium (DSS)-induced colitis in mice are due to its antioxidant and anti-inflammatory effects [40]. Likewise, another study demonstrates how the beneficial properties of the postbiotics ET-22 (Lacticaseibacillus paracasei) and BL-99 (Bifidobacterium lactis) on modulating the microbiota and inhibiting the pathogenic biofilm are also related to their anti-inflammatory and antioxidant effects [21,41,42].

The anti-inflammatory and antioxidant activity of BPL1™ HT is most likely related to its beneficial effects improving AngII-induced endothelial dysfunction since both conditions, oxidative stress and inflammation, are the main physio pathological processes involved in the development of endothelial dysfunction in the context of hypertension [43]. In addition, the antihypertensive effects of BPL1™ HT may also be related to its activity modulating the mRNA levels of AngII receptors in cardiovascular tissues. Particularly, mice infused with AngII showed an overexpression of At1r and a downregulation in the gene expression of At2r in arterial tissue, which was prevented by supplementation with the postbiotic. In this regard, it has been reported that the long-term activation of AT1R produces vascular damage and results in vascular remodeling, endothelial dysfunction, and hypertension [44]. On the contrary, the pharmacological activation of AT2R shows opposite effects, producing vasorelaxation, increased insulin sensitivity, and decreased inflammation, which results in protective effects [45].

Although there is evidence in the literature about the positive effects of probiotics on endothelial function [46,47], to our knowledge, this is the first study reporting the beneficial effects of a heat-treated postbiotic in the prevention of hypertension-induced endothelial dysfunction.

Another important finding of this study is that supplementation with BPL1™ HT produces cardioprotective effects. Particularly, mice infused with AngII and supplemented with the postbiotic BPL1™ HT showed increased heart contractility both in basal conditions and after subjecting the hearts to coronary ischemia–reperfusion (IR). Moreover, BPL1™ HT supplementation significantly reduced the protein levels of the pro-apoptotic caspase-8 and attenuated the IR-induced apoptosis of cardiomyocytes in myocardial tissue. These effects were associated with a downregulation in the gene expression of At1r and Nox-1 in cardiac tissue pointing, again, to an antioxidant effect. Several studies have described the cardioprotective effects of different probiotics [48]. Particularly, there is a study reporting the beneficial effects of probiotic supplementation in IR-induced apoptosis through decreased oxidative stress [49]. The cardioprotective effects found after the supplementation of AngII infused mice with BPL1™ HT for four weeks are in agreement with those found in other studies performed with other heat-inactivated strains such as L. reuteri GMNL-263, which significantly improves cardiac function in obese hamsters through the activation of the IGF1R/PI3K/Akt cell survival pathway [49] and in obese rats, in which it significantly increases the cardiac ejection fraction by reducing caspase-3-mediated myocardial apoptosis [50]. In addition, the same heat-treated strain of L. reuteri also exerted cardioprotective effects in rats with type 1 diabetes induced by streptozotocin injection through its anti-inflammatory and antiapoptotic effects [51,52]. Likewise, supplementation with heat-treated L. paracasei L-137 ameliorated LV diastolic dysfunction in rats with metabolic syndrome thanks to its anti-inflammatory effects [53]. However, in all these models, the cardiac damage was induced by a metabolic condition (obesity, diabetes, or metabolic syndrome), which may be considerably different than the cardiac alterations produced in the context of AngII-induced hypertension.

## 5. Conclusions

In conclusion, this study shows, for the first time, the beneficial cardiovascular effects of supplementation with the postbiotic BPL1™ HT on cardiovascular function in mice with hypertension induced by AngII infusion. Particularly, BPL1™ HT supplementation exerts antihypertensive effects and reduces IR-induced alterations on cardiac function. These effects are associated with a significant decrease in the gene expression of inflammation and oxidative stress markers in arterial and cardiac tissues, as well as with improved circulating levels of MDA. Thus, thanks to its antioxidant and anti-inflammatory effects, supplementation with BPL1™ HT could be an interesting approach in the management of cardiovascular health associated with the overstimulation of the RAS system.

## Figures and Tables

**Table 1:** Weights of heart, spleen, liver, epididymal visceral adipose tissue, retroperitoneal visceral adipose tissue, lumbar subcutaneous adipose tissue, interscapular brown adipose tissue, kidneys, adrenal glands, brain, and gastrocnemius of mice infused with saline (control), mice infused with AngII (AngII), and mice infused with AngII and supplemented with HTBPL-1 (AngII + HTBPL1).

Weight	Control	AngII	AngII + HTBPL1
Heart (mg/cm)	198.8 ± 9.5	248.9 ± 9.7 **	239.5 ± 14.6 ^#^
Spleen (mg/cm)	79.5 ± 4.8	77.8 ± 5.4	74.9 ± 3.5
Liver (mg/cm)	1219.1 ± 63	1152.4 ± 30	1144.6 ± 38.7
Epidydimal visceral adipose tissue (mg/cm)	423.2 ± 32.5	359.9 ± 35.7	373.3 ± 28.7
Retroperitoneal visceral adipose tissue (mg/cm)	100.5 ± 13.2	92.8 ± 10.5	115.6 ± 10.4
Lumbar subcutaneous adipose tissue (mg/cm)	189.1 ± 23	164.7 ± 20	164.1 ± 12
Interscapular brown adipose tissue (mg/cm)	105 ± 11.6	95.5 ± 10.8	53.6 ± 2.9
Kidneys (mg/cm)	286.6 ± 7.7	272.8 ± 12.1	254.5 ± 5.8 ^##^
Adrenal glands (mg/cm)	2.6 ± 0.2	3.2 ± 0.2 *	2.8 ± 0.2 *
Brain (mg/cm)	465.6 ± 4.4	465.6 ± 4.4	460.3 ± 5.4
Gastrocnemius (mg/cm)	139.2 ± 6.6	139.2 ± 6.6	141 ± 5.3

Data are represented as mean value ± SEM; *n* = 7–8 mice/group. * *p* < 0.05 vs. control; ** *p* < 0.01 vs. control; # *p* < 0.05 vs. AngII; ## *p* < 0.01 vs. AngII.

## Data Availability

Data are contained within the article.
